# Dysglycemias in patients admitted to ICUs with severe acute respiratory syndrome due to COVID-19 versus other causes - a cohort study

**DOI:** 10.1186/s12890-023-02439-y

**Published:** 2023-05-16

**Authors:** Rosângela Roginski Réa, Rafaella Stradiotto Bernardelli, Amanda Christina Kozesinski-Nakatani, Marcia Olandoski, Marcelo José Martins-Junior, Mirella Cristine Oliveira, Álvaro Réa-Neto

**Affiliations:** 1 Internal Medicine Department, Endocrine Division (SEMPR), Federal University of Paraná, Curitiba, Paraná Brazil; 2 Center for Studies and Research in Intensive Care Medicine (CEPETI), 366 Monte Castelo Street, Curitiba, Paraná 82590-300 Brazil; 3 School of Medicine and Life Sciences, Pontifical Catholic University of Paraná, Curitiba, Paraná Brazil; 4 ICU Department, Hospital Santa Casa de Curitiba, Curitiba, Paraná Brazil; 5 ICU Department, Complexo Hospitalar do Trabalhador (CHT), Curitiba, Paraná Brazil; 6 Internal Medicine Department, Federal University of Paraná, Curitiba, Paraná Brazil

**Keywords:** COVID-19, Critical illness, Intensive care units, Hyperglycemia, Glycemic control

## Abstract

**Background:**

Dysglycemias have been associated with worse prognosis in critically ill patients with COVID-19, but data on the association of dysglycemia with COVID-19 in comparison with other forms of severe acute respiratory syndrome are lacking. This study aimed to compare the occurrence of different glycemic abnormalities in patients with severe acute respiratory syndrome and COVID-19 admitted to intensive care units versus glycemic abnormalities in patients with severe acute respiratory syndrome from other causes, to evaluate the adjusted attributable risk associated with COVID-19 and dysglycemia and to assess the influence of these dysglycemias on mortality.

**Methods:**

We conducted a retrospective cohort of consecutive patients with severe acute respiratory syndrome and suspected COVID-19 hospitalized in intensive care units between March 11 and September 13, 2020, across eight hospitals in Curitiba-Brazil. The primary outcome was the influence of COVID-19 on the variation of the following parameters of dysglycemia: highest glucose level at admission, mean and highest glucose levels during ICU stay, mean glucose variability, percentage of days with hyperglycemia, and hypoglycemia during ICU stay. The secondary outcome was the influence of COVID-19 and each of the six parameters of dysglycemia on hospital mortality within 30 days from ICU admission.

**Results:**

The sample consisted of 841 patients, of whom 703 with and 138 without COVID-19. Comparing patients with and without COVID-19, those with COVID-19 had significantly higher glucose peaks at admission (165 mg/dL vs. 146 mg/dL; p = 0.002) and during ICU stay (242 mg/dL vs. 187md/dL; p < 0.001); higher mean daily glucose (149.7 mg/dL vs. 132.6 mg/dL; p < 0.001); higher percentage of days with hyperglycemia during ICU stay (42.9% vs. 11.1%; p < 0.001); and greater mean glucose variability (28.1 mg/dL vs. 25.0 mg/dL; p = 0.013). However, these associations were no longer statistically significant after adjustment for Acute Physiology and Chronic Health Evaluation II scores, Sequential Organ Failure Assessment scores, and C-reactive protein level, corticosteroid use and nosocomial infection. Dysglycemia and COVID-19 were each independent risk factors for mortality. The occurrence of hypoglycemia (< 70 mg/dL) during ICU stay was not associated with COVID-19.

**Conclusion:**

Patients with severe acute respiratory syndrome due to COVID-19 had higher mortality and more frequent dysglycemia than patients with severe acute respiratory syndrome due to other causes. However, this association did not seem to be directly related to the SARS-CoV-2 infection.

**Supplementary Information:**

The online version contains supplementary material available at 10.1186/s12890-023-02439-y.

## Background

In critically ill patients, the occurrence of glycemic abnormalities (dysglycemias) such as hyperglycemia, glycemic variability, and increased time out of target range (70 to 140 mg/dL) are associated with increased mortality in patients with and without diabetes [1–5]. Elevations in blood glucose levels are common in patients with severe acute respiratory syndrome (SARS) caused by SARS-CoV-2 infection (i.e., COVID-19) [6] and, even in individuals without diabetes, are associated with worse prognosis, longer stay in the intensive care unit (ICU), more frequent requirement of mechanical ventilation, and higher risk of hospital mortality [7–11]. Still, the literature assessing the association of dysglycemias with severe COVID-19 against other forms of SARS and associated outcomes remains scarce [12].

Based on these considerations, the primary aim of this cohort study was to compare the occurrence of different glycemic abnormalities in patients admitted to ICU with SARS and COVID-19 versus glycemic abnormalities in patients with SARS from other causes, to evaluate the adjusted attributable risk associated with COVID-19 and dysglycemia. The secondary aim was to determine the influence of these glycemic abnormalities on mortality. By comparing the progression of dysglycemia in patients with SARS due to COVID-19 against other causes, we sought to identify whether the dysglycemia would be specifically associated with the SARS-CoV-2 infection itself or merely related to the infection severity. We hope the findings will help guide research and care of future patients.

## Methods

This retrospective cohort study included consecutive patients with SARS and suspected COVID-19 admitted to ICUs across eight hospitals in the city of Curitiba (Brazil) between March 11 and September 13, 2020. During the study period, the combined maximum number of beds for patients with COVID-19 in these ICUs was 225. Of these, 124 were dedicated to patients covered by the Brazilian Unified Health System (SUS), 71 were for patients covered by health insurance plans or those paying their hospital bills, and 30 beds were for any patient regardless of health insurance coverage.

The study was approved by the local ethics committee of the *Instituto de Neurologia de Curitiba* under protocol number 2.899.188 on September 17, 2018 (ID: CAAE 98099918.2.0000.5227; project title: Epidemiological analysis of patients hospitalized in intensive care units in Curitiba-Paraná). The requirement for informed consent was waived by the same committee, given the noninterventional design of the study and the fact that the data were collected from clinical records and without contact with the participants and the procedures performed in this study were part of routine care. All research procedures were conducted in accordance with the ethical standards of the institutional committee on human experimentation and with the Helsinki Declaration of 1975 revised in 2013. The STROBE guidelines were used to ensure the reporting of this study.

The study included patients older than 18 years admitted to the ICUs with SARS due to strongly suspected or confirmed COVID-19, who had available result of a real-time polymerase chain reaction (RT-PCR) test for detection of SARS-CoV-2 collected by nasopharyngeal swab. Patients were classified according to suspicion through the set of clinical and radiological criteria for screening of COVID-19, which was routinely used in the study institutions during the pandemic period. They were considered to have SARS with strongly suspected COVID-19 when two of the following set of clinical-radiological criteria were present: (A) at least one flu-like symptom, i.e., cough, runny nose, fever, or sore throat; (B) at least two items from the modified quick SOFA scale (systolic blood pressure < 100 mmHg, respiratory rate > 22 bpm, decreased level of consciousness with Glasgow < 15, and oxygen pulse saturation < 93%); and (C) chest computed tomography (CT) scans with images suggestive of COVID-19 (ground-glass opacity and peripheral lesions distributed across both lungs) obtained in the first 48 h after admission [13, 14]. We excluded patients without complete medical records of daily follow-up during the ICU stay and those without records of venous or capillary glucose values on at least 40% of the ICU days.

An electronic database was created in REDCap (Vanderbilt University, Nashville, TN, USA) for daily and systematic recording and sharing of the data collecting form among the group researchers. The data, collected from all study patients, included demographic characteristics, medical history, and daily clinical and laboratory information related to the first 30 days in the ICU or until the outcome of discharge or death, whichever occurred first.

The highest and lowest daily values of capillary and venous blood glucose were used to calculate the parameters of glucose abnormalities during ICU as follows: highest glucose level on admission, corresponding to the highest glucose value recorded in the first 24 h in the ICU; highest glucose level during ICU stay, corresponding to the highest glucose level recorded during the entire ICU stay; mean glucose level during ICU stay, calculated daily and individually from the mean glucose level of each day in the ICU, subsequently added up and divided by the number of days in which glucose was recorded during ICU stay; mean glucose variability during ICU stay, as reflected by mean of daily glycemic coefficient of variation (CV) during ICU stay; the percentage of days with hyperglycemia (≥ 180 mg/dL), calculated as the number of days with any glycemic value ≥ 180 mg/dL divided by the number of ICU days in which glycemia was recorded and multiplied by 100; and the occurrence of hypoglycemia (< 70 mg/dL) during ICU stay, which represents the number of patients who had at least one glycemic value < 70 mg/dL during the ICU stay period.

**Fig. 1:**
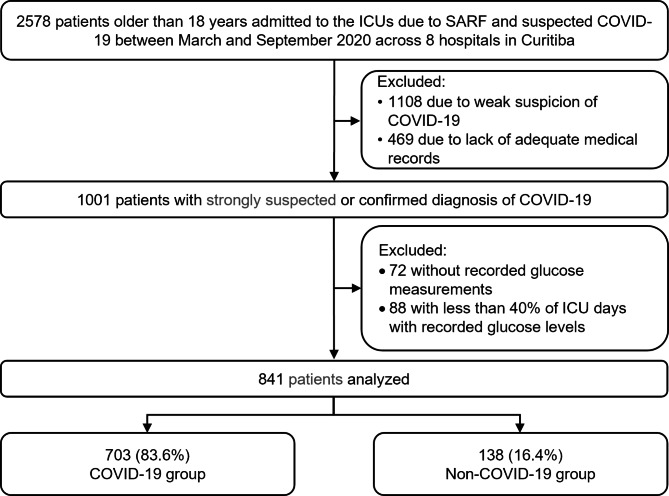
Process of selection of the study sample

Glucose variability was calculated from capillary blood samples analyzed with a glucometer, while all other parameters of glucose abnormalities were measured in capillary or venous blood samples. Although glucose values are on average 10.19% higher when measured in capillary compared with venous blood, the proportions of patients with glucose values measured in venous blood were not different between groups.

### Glucose control

In the present study, hyperglycemia was defined as blood glucose level > 180 mg/dl and hypoglycemia as blood glucose value < 70 mg/dl. According to the treatment protocol followed by all hospitals involved in the study, intravenous insulin infusion was initiated after the first measurement of blood glucose higher than 180 mg/dl. Then, the following measurements were performed every 2 h, and insulin infusion was adjusted according to the new value found, as follow: insulin drip was stopped when blood glucose was < 80 mg/dl, it was decreased by 2UI/ml/h if it was between 81 and 110, between 111 and 180 no change of rate was made, and if the blood glucose was > 180, insulin drip was increased by 2UI/ml/h.

The patients were divided into a group with a diagnosis of COVID-19 confirmed by RT-PCR (COVID-19 group) and another group in whom this diagnosis was refuted (non-COVID-19 group). To mitigate the bias of false-negative test results, the non-COVID-19 group included either patients who presented more than one negative RT-PCR result or with only one negative RT-PCR result, if the patient had another cause most likely to explain the diagnosis of SARS than SARS-CoV-2 infection. The diagnosis of patients who comprised the non-COVID-19 group are described in the Results section.

**Table 1:** Characteristics of the overall cohort and the COVID-19 and non-COVID-19 groups

Variables	Overall cohort (n = 841)	COVID-19 group (n = 703)	Non-COVID-19 group (n = 138)	p value
**Baseline**				
Age, years	61 ± 16.6	60.6 ± 15.8	63.5 ± 19.7	0.052†
Male sex	371 (44.1)	302 (43)	69 (50)	0.134*
**Comorbidities**				
Charlson Comorbidity Index	1 (0–2)	1 (0–2)	1 (0–2)	0.001^#^
History of diabetes	259 (30.8)	222 (31.6)	37 (26.8)	0.313*
Hypertension	436 (51.8)	369 (52.5)	67 (48.6)	0.403*
Cardiopathy	156 (18.5)	121 (17.2)	32 (25.4)	0.031*
Chronic obstructive pulmonary disease	105 (12.5)	69 (9.8)	36 (26.1)	< 0.001*
Renal disease	50 (5.9)	44 (6.4)	4 (4.3)	0.554*
Peripheral vascular diseases	20 (2.4)	18 (2.6)	2 (1.4)	0.758*
Cerebrovascular diseases	40 (4.8)	28 (4.0)	12 (8.7)	0.027*
Chronic neurologic diseases	34 (4.0)	23 (3.3)	11 (8.0)	0.017*
Cognitive impairment or dementia	31 (3.7)	17 (2.4)	14 (10.1)	< 0.001*
Rheumatologic or connective tissue disease	28 (3.3)	26 (3.7)	2 (1.4)	0.295*
Chronic liver disease	10 (1.2)	7 (1.0)	3 (2.2)	0.217*
Cancer	33 (3.9)	27 (3.8)	6 (4.3)	0.810*
HIV/AIDS	11 (1.3)	9 (1.3)	2 (1.4)	0.699*
Presumed obesity	250 (29.7)	226 (32.1)	24 (17.4)	< 0.001*
**ICU admission status**				
Time from symptom onset to ICU admission, days^a^	7 (4–9)	7 (5–10)	4 (2–7)	< 0.001^#^
APACHE II score in the first 24 h in ICU	13 (8–19)	13 (8–20)	13 (8–18)	0.707^#^
SOFA score in the first 24 h in ICU	4 (2–6)	3 (2–6)	4 (2–6)	0.350^#^
CRP level in the first 24 h in the ICU^b^	115 (57.1–177)	23 (68.9–184)	43.1 (15–124)	< 0.001^#^
VAD at admission	144 (17.1)	119 (16.9)	25 (18.1)	0.712*
IMV at admission	232 (27.6)	191 (27.2)	41 (29.7)	0.534*
**Related to glycemic abnormalities in the ICU**				
Highest glucose value at ICU admission^c^ (mg/dL)	161(123–224)	165 (124–235)	146 (118–187)	0.002 ^#^
Highest glucose value during ICU stay (mg/dL)	233 (167–372)	242 (172–383)	187 (142–289)	< 0.001^#^
Mean glucose value during ICU stay (mg/dL)	145.9 (121.5–196.8)	149.7 (122.9–201)	132.6 (111.6–171.6)	< 0.001^#^
Mean glucose variability (CV %) during ICU stay^d^	27.4 (20.2–39.9)	28.1 (20.4–41.1)	25.0 (18.7–34.5)	0.013^#^
% of days with hyperglycemia (≥ 180 mg/dL)	33.3 (0–82.4)	42.9 (0–85.7)	11.1 (0–66.7)	< 0.001^#^
Occurrence of hypoglycemia (< 70 mg/dL) during ICU stay	218 (25.9)	184 (26.2)	34 (24.6)	0.751*
**Related to progression within 30 days of ICU stay**				
Nosocomial infection	169 (20.1)	151 (21.5)	18 (13)	0.027*
Mean SOFA score during ICU stay	4.3 (2.3–7)	4.4 (2.3–7.3)	4.1 (2.2–5.9)	0.033^#^
Mean CRP level during ICU stay^b^	118.7 (65.3–178.7)	126.5 (73.9–182.7)	66.4 (24–144.6)	< 0.001^#^
Use of VAD during ICU stay	423 (50.3)	370 (52.6)	53 (38.4)	0.003*
Use of IMV during ICU stay	445 (52.9)	385 (54.8)	60 (43.5)	0.016*
Lowest PaO2/FiO2 ratio during ICU stay				< 0.001‡
> 300	72 (8.6)	54 (7.7)	18 (13)	
≤ 300	164 (19.5))	120 (17.1)	44 (31.9)	
≤ 200	258 (30.7)	208 (29.6)	50 (36.2)	
≤ 100	347 (41.3)	321 (45.7)	26 (18.8)	
Use of corticosteroid during ICU stay	551 (65.5)	499 (71)	52 (37.7)	< 0.001*
ICU length of stay	6 (3–12)	6 (3–13)	4.5 (3–10)	0.011^#^
ICU mortality	320 (38)	290 (41.3)	30 (21.7)	< 0.001*

Data are expressed as number (percent), mean ± standard deviation, or median (first quartile - third quartile)

Abbreviations: APACHE II, Acute Physiology and Chronic Health Evaluation II; SOFA, Sequential Organ Failure Assessment; CRP, C-reactive protein (mg/L); VAD: vasoactive drug; IMV: invasive mechanical ventilation. The glucose values are shown as mg/dL; hrs: hours; CV, Coefficient of variation

Missing data: ^a^105 in the overall cohort, 84 in the COVID-19 group, and 21 in the non-COVID-19 group; ^b^4 in the overall cohort, 4 in the COVID-19 group, and none in the non-COVID-19 group; ^c^4 in the overall cohort, 3 in the COVID-19 group, and 1 in the non-COVID-19 group; ^d^76 in the overall cohort, 49 in the COVID-19 group, and 27 in the non-COVID-19 group

*Fisher’s exact test

^†^Student’s t test for independent samples

^#^Nonparametric Mann-Whitney test

^‡^Chi-square test

p < 0.05 indicates statistical significance

We evaluated the influence of COVID-19 on the variation of the six parameters of glycemic abnormalities described as the primary outcome. As the secondary outcome, we evaluated the influence of COVID-19 and each of the six parameters of glycemic abnormalities on hospital mortality within 30 days of ICU admission.

Following recommendations for observational studies in critically ill patients [15], we determined *a priori* the confounding factors of the relationship between the dysglycemias and the outcomes, based on the probability of association of these confounders both with COVID-19 and with the parameters of glycemic abnormality [16]. Thus, we considered as possible confounders or modifiers of the effect of COVID-19 on the outcomes the following variables: sex; age; prior history of diabetes [9, 17]; presumed obesity; Charlson Comorbidity Index; Acute Physiology and Chronic Health Evaluation (APACHE) II scores, Sequential Organ Failure Assessment (SOFA) scores [18], and C-reactive protein (CRP) values in the first 24 h in the ICU and the mean values and scores during the entire ICU stay [19, 20]; presence of nosocomial infection (presence of bacterial infection after 48 h in the ICU and antibiotic therapy that continued for at least 72 h) [21]; and use of corticosteroids, defined as the use of corticosteroids on admission or started within 24 h, of admission and maintained during hospitalization, initially hydrocortisone 200 mg daily. After the preliminary results of the RECOVERY study were released, dexamethasone 6 mg daily was the standard corticosteroid therapy [22, 23]. Such variables were also considered as possible confounders or modifiers of the independent effect of COVID-19 and glycemic abnormalities on mortality.

**Table 2:** Influence of COVID-19 on each of the parameters of glycemic abnormality unadjusted and adjusted for the variables sex, history of diabetes, presumed obesity, age, APACHE II scores, SOFA scores, CRP levels, and corticosteroid use

Factors associated with highest glucose level on admission	n^$^	e ^Coef^. (95% CI) unadjusted*	p value^§^	n^$$^	e ^Coef^. (95% CI) adjusted**	p value^§^
COVID-19 ^a^	837	1.144 (1.054–1.241)	0.001	833	1.034 (0.959–1.115)	0.380
Male sex ^b^	837	0.939 (0.884–0.999)	0.046		0.981 (0.93–1.035)	0.489
History of diabetes ^c^	837	1.518 (1.43–1.611)	< 0.001		1.479 (1.395–1.568)	0.000
Presumed obesity ^d^	837	1.084 (1.015–1.159)	0.017		1.032 (0.973–1.094)	0.293
Charlson Comorbidity Index	837	1.036 (1.013–1.059)	0,002		-	-
Age	837	1.002 (1–1.003)	0.080		0.997 (0.996–0.999)	0.003
APACHE II scores	837	1.012 (1.009–1.015)	< 0.001		1.008 (1.005–1.012)	0.000
SOFA scores in the first 24 h	837	1.034 (1.023–1.046)	< 0.001		1.017 (1.005–1.028)	0.005
CRP in the first 24 h	833	1.001 (1.001–1.001)	< 0.001		1.000 (1.000–1.001)	0.039
Use of corticosteroids ^f^	837	1.180 (1.108–1.257)	< 0.001		1.163 (1.099–1.231)	0.000
					*BIC*: 838.168‡	
**Factors associated with highest glucose levels during hospitalization**	**n** ^**$**^	**e** ^**Coef.**^ (95% CI) unadjusted*	**p value** ^**§**^	**n** ^**$$**^	**e** ^**Coef.**^ (95% CI) adjusted**	**p value** ^**§**^
COVID-19 ^a^	841	1.237 (1.133–1.35)	< 0.001	837	1.067 (0.995–1.145)	0.069
Male sex ^b^	841	0.959 (0.898–1.025)	0.217		-	-
History of diabetes ^c^	841	1.583 (1.485–1.688)	< 0.001		1.524 (1.444–1.609)	0.000
Presumed obesity ^d^	841	1.177 (1.096–1.264)	< 0.001		1.098 (1.039–1.161)	0.001
Charlson Comorbidity Index	841	1.061 (1.035–1.086)	< 0.001		-	-
Age	841	1.004 (1.002–1.006)	< 0.001		0.999 (0.998–1.001)	0.369
APACHE II scores	841	1.014 (1.011–1.018)	< 0.001		1.003 (1–1.006)	0.060
Nosocomial infection ^e^	841	1.57 (1.455–1.695)	< 0.001		1.376 (1.29–1.468)	0.000
Mean SOFA during ICU stay	841	1.068 (1.058–1.079)	< 0.001		1.045 (1.032–1.057)	0.000
Mean CRP during ICU stay	837	1.002 (1.001–1.002)	< 0.001		1.000 (1.000–1.000)	0.745
Use of corticosteroids ^f^	837	1.211 (1.131–1.296)	< 0.001		1.172 (1.111–1.236)	0.000
					*BIC*: 741.262‡	
**Factors associated with mean glycemic levels during hospitalization**	**n** ^**$**^	**e** ^**Coef.**^ (95% CI) unadjusted*	**p value** ^**§**^	**n** ^**$$**^	**e** ^**Coef.**^ (95% CI) adjusted**	**p value** ^**§**^
COVID-19 ^a^	841	1.121 (1.054–1.191)	< 0.001	837	1.029 (0.977–1.084)	0.284
Male sex ^b^	841	0.977 (0.933–1.023)	0.312		-	-
History of diabetes ^c^	841	1.432 (1.371–1.495)	< 0.001		1.398 (1.342–1.455)	< 0.001
Presumed obesity ^d^	841	1.123 (1.069–1.18)	< 0.001		1.078 (1.035–1.123)	< 0.001
Charlson Comorbidity Index	841	1.044 (1.027–1.062)	< 0.001		-	-
Age	841	1.003 (1.001–1.004)	< 0.001		0.999 (0.998–1.001)	0.267
APACHE II	841	1.009 (1.007–1.012)	< 0.001		1.004 (1.001–1.006)	0.003
Nosocomial infection ^e^	841	1.185 (1.121–1.254)	< 0.001		1.101 (1.049–1.155)	< 0.001
Mean SOFA during ICU stay	841	1.035 (1.028–1.043)	< 0.001		1.02 (1.011–1.029)	< 0.001
Mean CRP during ICU stay	837	1.001 (1.001–1.001)	< 0.001		1.000 (1.000–1.000)	0.466
Use of corticosteroids ^f^	837	1.104 (1.053–1.158)	< 0.001		1.089 (1.047–1.134)	< 0.001
					*BIC*: 252.794‡	
**Factors associated with % of days with hyperglycemia (≥ 180 mg/dL)**	**n** ^**$**^	**Coef. (95% CI) unadjusted** ^**#**^	**p value** ^**§**^	**n** ^**$$**^	**Coef. (95% CI) adjusted** ^**##**^	**p value** ^**§**^
COVID-19 ^a^	841	13.528 (6.44–20.616)	< 0.001	837	3.233 (-2.813–9.28)	0.295
Male sex ^b^	841	-3.805 (-9.13–1.519)	0.161		0.176 (-4.178–4.53)	0.937
History of diabetes ^c^	841	41.881 (36.895–46.867)	< 0.001		39.565 (34.869–44.261)	0.000
Presumed obesity ^d^	841	9.123 (3.364–14.881)	0.002		4.271 (-0.483–9.026)	0.078
Charlson Comorbidity Index	841	5.365 (3.437–7.294)	< 0.001		-	-
Age	841	0.328 (0.17–0.487)	< 0.001		-0.051 (-0.196–0.094)	0.490
APACHE II scores	841	1.036 (0.774–1.298)	< 0.001		0.356 (0.075–0.636)	0.013
Nosocomial infection ^e^	841	20.309 (13.848–26.77)	< 0.001		12.01 (6.443–17.576)	0.000
Mean SOFA during ICU stay	841	3.965 (3.121–4.808)	< 0.001		2.164 (1.131–3.197)	0.000
Mean CRP during ICU stay	837	0.107 (0.074–0.139)	< 0.001		0.023 (-0.009–0.055)	0.163
Use of corticosteroid ^f^	837	12.994 (7.495–18.493)	< 0.001		12.054 (7.443–16.665)	0.000
					*BIC*: 8208.738‡	
**Factors associated with mean glucose variability (CV %) during ICU stay**	**n** ^**$**^	**Coef. (95% CI) unadjusted** ^**#**^	**p value** ^**§**^	**n** ^**$$**^	**Coef. (95% CI) adjusted** ^**##**^	**p value** ^**§**^
COVID-19 ^a^	765	4.243 (1.033–7.454)	0.010	762	1.999 (-0.773–4.771)	0.158
Male sex ^b^	765	-3.114 (-5.392 - -0.837)	0.007		-0.716 (-2.642–1.21)	0.466
History of diabetes ^c^	765	16.237 (14.071–18.404)	< 0.001		15.04 (12.978–17.102)	< 0.001
Presumed obesity ^d^	765	-1.634 (-4.114–0.847)	0.202		-	-
Charlson Comorbidity Index	765	3.279 (2.44–4.119)	< 0.001		-	-
Age	765	0.204 (0.136–0.272)	< 0.001		0.011 (-0.053–0.076)	0.730
APACHE II scores	765	0.527 (0.413–0.64)	< 0.001		0.252 (0.127–0.377)	< 0.001
Nosocomial infection ^e^	765	4.282 (1.532–7.033)	0.002		1.186 (-1.216–3.588)	0.333
Mean SOFA during ICU stay	765	1.678 (1.303–2.052)	< 0.001		1.012 (0.545–1.479)	< 0.001
Mean CRP during ICU stay	762	0.033 (0.019–0.047)	< 0.001		-0.003 (-0.017–0.012)	0.722
Use of corticosteroid ^f^	765	4.197 (1.744–6.65)	< 0.001		4.098 (2.018–6.178)	< 0.001
					BIC: 6153.797‡	

Abbreviations: APACHE II, Acute Physiology and Chronic Health Evaluation II; SOFA: Sequential Organ Failure Assessment; CRP: C-reactive protein (mg/L)

Reference categories: ^a^non-COVID-19; ^b^female sex; ^c^absence of history of known diabetes; ^d^absence of presumed obesity; ^e^absence of nosocomial infection; ^f^no use of corticosteroids during ICU stay

*Exponentiated linear regression coefficient (**e**^**Coef.**^) and 95% confidence interval (95% IC) of the univariate log-linear regression model

**Exponentiated linear regression coefficient (**e**^**Coef.**^) and 95% confidence interval (95% IC) of the multivariate log-linear regression model

^#^Linear regression coefficient (Coef.) and 95% confidence interval (95% IC) of the univariate log-linear regression model

^##^Linear regression coefficient (Coef.) and 95% confidence interval (95% IC) of the multivariate log-linear regression model

^$^number of cases (n) included in each univariate model. ^$$^number of cases (n) included in the multivariate model

^§^Wald test p value, p < 0.05 indicates statistical significance

^‡^Bayesian information criterion (BIC) of the multivariate generalized linear model, representative of the quality of the model given its explanatory potential, with the lower the BIC, the more the model is able to explain the dependent variable

### Statistical analysis

Categorical variables are presented as frequencies and percentages. The normality of the distribution was verified visually using box plots, skewness and kurtosis assessments, and the Kolmogorov-Smirnov test. Quantitative variables with normal distribution are presented as means and standard deviations, and those with non-normal distribution as medians and interquartile ranges. Frequencies were compared using the chi-square test or Fisher’s exact test, as appropriate. Quantitative variables were compared between groups using Student’s *t* test for independent samples when the data were normally distributed or the nonparametric Mann-Whitney test when the data were not normally distributed.

**Table 3:** Significance of the dysglycemias variables comparison between COVID-19 and non-COVID-19, restricted by corticosteroids’ use or no use

Variables	Use of corticosteroids	No use of corticosteroids
	p value (COVID-19 vs. non-COVID-19)	p value (COVID-19 vs. non-COVID-19)
Highest glucose level at ICU admission	0.157*	0.967*
Highest glucose level during ICU stay	0.151^#^	0.234^#^
Mean glucose level during ICU stay	0.203^#^	0.741^#^
Mean glucose variability (CV %) during ICU stay	0.793^†^	0.078^†^
% of days with hyperglycemia (≥ 180 mg/dL)	0.713^§^	0.277^§^

*Wald test p value of the multivariate log-linear regression models adjusted for sex, history of diabetes, presumed obesity, age, APACHE II scores, SOFA scores in the first 24 h, and CRP levels in the first 24 h

^#^Wald test p value of the multivariate log-linear regression models adjusted for history of diabetes, presumed obesity, age, APACHE II scores, Nosocomial infection, Mean SOFA during ICU stay, Mean CRP during ICU stay

^†^Wald test p value of the multivariate linear regression models adjusted for sex, history of diabetes, age, APACHE II scores, Nosocomial infection, Mean SOFA during ICU stay, Mean CRP during ICU stay

^§^Wald test p value of the multivariate linear regression models adjusted for sex, history of diabetes, presumed obesity, age, APACHE II scores, Nosocomial infection, Mean SOFA during ICU stay, Mean CRP during ICU stay

We fitted univariate and multivariate generalized linear models with log(n)-transformed data and linear scale response using Wald parameter estimates to assess the explanatory potential of COVID-19, sex, diabetes history, presumed obesity, Charlson Comorbidity Index, age, APACHE II scores, SOFA scores, CRP levels, corticosteroid use, and presence of nosocomial infection for each of the hyperglycemic parameters: highest glucose value at admission; highest glucose value during ICU stay; mean glucose level during ICU stay, and mean glucose variability during ICU stay. The same models were used without log(n)-transformed data to evaluate the glycemic abnormality “percentage of days with hyperglycemia.“ The significance of each model’s variables was evaluated using the Wald test. The results of the log-linear models are presented as exponentiated linear regression coefficients (e^Coef^.) and 95% confidence intervals (95% CIs), while the results of the linear models are presented as linear regression coefficients (Coef.) and 95% CIs.

**Table 4:** Risk factors for 30 days ICU mortality of patients admitted for SARS with or without COVID-19

Factors		Mortality in the overall cohort		OR (95% CI)	p value^§^
		**n** ^†^	**Results** ^**#**^		
Diagnosis	Non-COVID-19	30	30 (21.7)	Ref.	
	COVID-19	290	290 (41.3)	2.528 (1.642–3.892)^*^	< 0.001
Sex	Female	371	144 (38.8)	Ref.	
	Male	470	176 (37.4)	0.911 (0.686–1.211)^**^	0.522
History of diabetes	No	582	207 (35.6)	Ref.	
	Yes	259	113 (43.6)	1.377 (1.018–1.861)^**^	0.038
Presumed obesity	No	591	232 (39.3)	Ref.	
	Yes	250	88 (35.2)	0.770 (0.563–1.052)^**^	0.101
Charlson Comorbidity Index	No death	521	1 (0–1)		
	Death	320	1 (0–2)	1.300 (1.167–1.447)	< 0.001
Age	No death	521	57.6 ± 16.7		
	Death	320	66.7 ± 14.7	1.041 (1.030–1.051)^**^	< 0.001
APACHE II in the first 24 h	No death	521	10 (6–15)		
	Death	320	19 (12–27)	1.123 (1.101–1.146)^**^	< 0.001
SOFA scores in the first 24 h	No death	521	3 (2–4)		
	Death	320	6 (3–8)	1.524 (1.423–1.633)^**^	< 0.001
CRP levels in the first 24 h	No death	518	91.6 (39–156)		
	Death	319	142 (88–219)	1.006 (1.004–1.008)^**^	< 0.001
Highest glucose level at admission(mg/dL)	No death	520	150.5 (118–199.5)		
	Death	317	180 (136–290)	1.005 (1.004–1.007)^**^	< 0.001
Highest glucose level during ICU stay (mg/dL)	No death	521	196 (156–294)		
	Death	320	335 (209.5–453.5)	1.006 (1.005–1.007)^**^	< 0.001
Mean glucose level during ICU stay (mg/dL)	No death	521	133.6 (115.5–166.3)		
	Death	320	178.7 (138.5–226.9)	1.012 (1.009–1.015)^**^	< 0.001
Mean glucose variability (CV %) during ICU stay	No death	469	23.9 (18.2–33.1)		
	Death	296	35.5 (25.0–46.8)	1.041 (1.030–1.052)	< 0.001
% of days with hyperglycemia	No death	521	17.6 (0–60)		
	Death	320	66.7 (28.6–93.3)	1.018 (1.014–1.022)^**^	< 0.001
Occurrence of hypoglycemia (< 70 mg/dL) during ICU stay	No	623	197 (31.6)	Ref.	
	Yes	218	123 (56.4)	2.848 (2.065–3.927)	< 0.001
Nosocomial infection	No	672	211 (31.4)	Ref.	
	Yes	169	109 (64.5)	3.841 (2.685–5.493)^**^	< 0.001
Mean SOFA score during ICU stay	No death	521	2.8 (2–4.1)		
	Death	320	7.6 (6–9.2)	2.786 (2.432–3.192)^**^	< 0.001
Mean CRP level during ICU stay	No death	518	83.2 (43.9–135.9)		
	Death	319	175.7 (126.9–224.9)	1.018 (1.015–1.020)^**^	< 0.001
Use of corticosteroids during ICU stay	No	290	95 (32.8)	Ref.	
	Yes	551	225 (40.8)	1.215 (0.891–1.655)^**^	0.218

Abbreviations: APACHE II, Acute Physiology and Chronic Health Evaluation II; SOFA, Sequential Organ Failure Assessment; CRP, C-reactive protein (mg/L)

^†^Total number of cases in the row

^#^Data are expressed as number (percent), mean ± standard deviation, or median (first quartile - third quartile)

*Odds ratio and 95% confidence interval of the generalized linear model with univariate binary logistic distribution for mortality during ICU stay up to 30 days

**Odds ratio and 95% confidence interval of the generalized linear model with binary logistic distribution for mortality during ICU stay up to 30 days, adjusted for the presence of COVID-19 (crude model)

^§^Wald test p value, p < 0.05 indicates statistical significance

The generalized linear models with binary logistic distribution using hybrid parameter estimation and fixed value scale were used to estimate the explanatory potential of COVID-19, sex, diabetes history, presumed obesity, Charlson Comorbidity Index, age, APACHE II scores, SOFA scores, CRP levels, corticosteroid use, presence of nosocomial infection and occurrence of hypoglycemia (< 70 mg/dL) during ICU stay, with results presented as Odds ratio (OR), 95% CI, and statistical significance of the Wald test.

Variables with significance less than 0.200 in the Wald test in the univariate analysis were included in the multivariate analysis. In cases where both diabetes history and the Charlson Comorbidity Index met this criterion, the Charlson Comorbidity Index was not included in the multivariable model given that diabetes is contained in the index result and therefore should not be adjusted in the same multivariable model. Using the same adjusted models mentioned above, we also performed an association analysis of COVID-19 with glycemic abnormalities variables stratified by use of corticosteroids during ICU stay for groups using corticosteroids against those not using corticosteroids. Generalized linear models with binary logistic distribution using hybrid parameter estimation and fixed value scale were used to estimate the explanatory potential of each of the parameters representing glucose abnormality and the variables COVID-19, sex, history of diabetes, presumed obesity, Charlson Comorbidity Index, age, APACHE II scores, SOFA scores, CRP levels, use of corticosteroids, and presence of nosocomial infection on mortality during ICU stay up to 30 days from admission. Odds ratio (OR), 95% CI, and statistical significance of the Wald test in the univariate model are presented for COVID-19, while for the other variables, the results of crude models that had COVID-19 as a covariate are presented. The ORs and 95% CIs of COVID-19 and each of the six variables of glycemic abnormality for mortality were adjusted, using multiple models, for the potential confounders established *a priori*. A p value < 0.05 was considered to indicate statistical significance.

The quality of the adjustments of the multiple models, given by their explanatory potential, was evaluated by the Bayesian information criterion (BIC). The statistical significance level was set at 5%, and data were analyzed using the statistical software IBM SPSS Statistics, version 28.0 (IBM SPSS Inc., Chicago, IL, USA). Missing data were not imputed.

**Table 5:** Adjusted models explaining mortality within 30 days in the ICU

Multivariate explanatory models of mortality in the overall cohort	n^#^	OR (95% CI) adjusted*	p value ^§^	BIC ‡
Highest glucose level at admission	833	1.004 (1.002–1.006)	< 0.001	847.026
COVID-19 (ref: non-COVID-19)		2.990 (1.767–5.060)	< 0.001	
History of diabetes (ref: no history)		0.658 (0.435–0.995)	0.048	
Age		1.026 (1.013–1.038)	< 0.001	
APACHE II score		1.057 (1.032–1.082)	< 0.001	
SOFA score in the first 24 h		1.299 (1.201–1.405)	< 0.001	
CRP level in the first 24 h		1.004 (1.002–1.006)	< 0.001	
Highest glucose level during ICU stay	837	1.003 (1.000–1.005)	0.021	519.168
COVID-19 (ref: non-COVID-19)		2.655 (1.363–5.17)	0.004	
History of diabetes (ref: no history)		0.816 (0.458–1.457)	0.493	
Age		1.020 (1.003–1.037)	0.023	
APACHE II score		0.997 (0.966–1.028)	0.847	
Nosocomial infection (ref: no infection)		0.976 (0.558–1.707)	0.933	
Mean SOFA score		2.327 (1.993–2.717)	< 0.001	
Mean CRP level		1.010 (1.006–1.013)	< 0.001	
Mean glucose level during ICU stay	837	1.011 (1.006–1.016)	< 0.001	502.875
COVID-19 (ref: non-COVID-19)		2.665 (1.336–5.315)	0.005	
History of diabetes (ref: no history)		0.566 (0.312–1.029)	0.062	
Age		1.024 (1.006–1.041)	0.009	
APACHE II score		0.993 (0.962–1.026)	0.684	
Nosocomial infection (ref: absent)		1.080 (0.630–1.851)	0.779	
Mean SOFA score		2.348 (2.014–2.737)	< 0.001	
Mean CRP level		1.01 (1.006–1.014)	< 0.001	
% of days with hyperglycemia (≥ 180 mg/dL)	837	1.017 (1.010–1.025)	< 0.001	504.526
COVID-19 (ref: non-COVID-19)		2.350 (1.192–4.633)	0.014	
History of diabetes (ref: no history)		0.555 (0.302–1.019)	0.058	
Age		1.020 (1.003–1.038)	0.020	
APACHE II score		0.993 (0.962–1.025)	0.669	
Nosocomial infection (Ref: absent)		1.010 (0.588–1.734)	0.971	
Mean SOFA score		2.370 (2.032–2.765)	< 0.001	
Mean CRP level		1.010 (1.006–1.014)	< 0.001	
Mean glucose variability (CV %) during ICU stay	762	1.032 (1.013–1.051)	0.001	476.005
COVID-19 (ref: non-COVID-19)		3.126 (1.503–6.502)	0.002	
History of diabetes (ref: no history)		0.651 (0.347–1.222)	0.181	
Age		1.022 (1.004–1.04)	0.015	
APACHE II score		0.984 (0.951–1.017)	0.329	
Nosocomial infection (ref: no infection)		1.235 (0.716–2.13)	0.449	
Mean SOFA score		2.444 (2.079–2.873)	0.000	
Mean CRP level		1.010 (1.006–1.014)	0.000	

Abbreviations: APACHE II, Acute Physiology and Chronic Health Evaluation II; SOFA, Sequential Organ Failure Assessment; CRP, C-reactive protein (mg/L)

^#^Case numbers included in the model

* Odds ratio (OR) and 95% confidence interval (95% CI) for mortality within 30 days in the ICU, generalized linear models with multivariate binary logistic regression

^§^Wald test p value, with values < 0.05 indicating statistical significance

^‡^Bayesian information criterion (BIC) of the multivariate generalized linear model representing the quality of the model given its explanatory potential; the lower the BIC, the more the model is able to explain the dependent variable

## Results

From March to September 2020, a total of 2578 patients older than 18 years were admitted to the participating ICUs with SARS due to strongly suspected or confirmed COVID-19. We excluded 1108 patients not meeting the inclusion criteria of strongly suspected COVID-19, 469 patients due to the absence of medical records of daily ICU follow-up, 72 without records of glucose measurements, and 88 with glucose values recorded in less than 40% of the days in the ICU. Thus, we analyzed data from 841 patients, of whom 83.6% (703) comprised the COVID-19 group and 16.4% (138) comprised the non-COVID-19 group (Fig. 1).

The non-COVID-19 group (n = 138) included the following diagnosis established to explain the diagnosis of acute respiratory failure: 54.3% had pneumonia; 18.1% cardiovascular diseases; 15.2% exacerbated chronic pneumopathies (asthma, COPD or pulmonary fibrosis); 3.6% sepsis of extrapulmonary etiology; 2.4% lung cancer; 2.2% neurological diseases; 1.5% pulmonary thromboembolism; 1.5% pneumonitis; 1.5% metabolic decompensation.

Table 1 presents the characteristics and outcomes of the entire cohort and each group between admission and 30 days of ICU stay, including the results of the six parameters of glycemic abnormalities analyzed. Patients with COVID-19 had significantly higher peaks of glucose values at admission and during ICU stay, higher mean daily glucose values, higher percentage of days with hyperglycemia during ICU stay, and greater mean daily glucose variability. However, there was no significant difference in the proportion of patients with an episode of hypoglycemia (< 70 mg/dL) during the ICU stay.

The characteristics of patients from each participating hospital are presented separately in Additional File 1: Table S1.

The COVID-19 group had a higher percentage of patients with presumed obesity (as determined by the attending physician), higher CRP values through ICU stay, higher mean SOFA score during ICU stay, higher incidence of nosocomial infection, and need for vasoactive drug and invasive mechanical ventilation. Considering the first 30 days of ICU stay, patients with COVID-19 stayed longer in the ICU and had a higher mortality rate in the period (Table 1).

Given differences between the groups regarding characteristics potentially influencing the causal relationship of COVID-19 and glucose abnormalities, the influence of COVID-19 on each of five of the continuous variables representing glucose abnormality at admission and during ICU stay were evaluated separately and adjusted for sex, history of diabetes, presumed obesity, age, APACHE II score, SOFA score, CRP level, presence of nosocomial infection, and use of corticosteroid. The results of the unadjusted and adjusted models are presented in Table 2.

COVID-19, history of diabetes, presumed obesity, age, APACHE II scores, use of corticosteroid, presence of nosocomial infection, SOFA scores, CRP values in the first 24 h, and mean values during ICU stay were each individually associated with the highest glucose level at admission, mean and highest glucose levels during ICU stay, mean glucose variability, and percentage of days with hyperglycemia, as shown in Table 2. However, the relationship between COVID-19 and these five parameters of glycemic abnormalities was no longer statistically significant after adjustment for confounders. On the other hand, APACHE II score, SOFA score, use of corticosteroids, and presence of nosocomial infection remained associated with these parameters representing glycemic abnormality, even after adjustments in the multivariate models (Table 2). The COVID-19 was not associated to the occurrence of hypoglycemia (> 70 mg/dL) during ICU stay nor in univariate analysis, nor after adjustments in the multivariate model (Additional file: Table S2)

The lack of association of COVID-19 with the six parameters of glycemic abnormalities was also confirmed in models restricted for use and no use of corticosteroids and adjusted for sex, history of diabetes, presumed obesity, age, APACHE II score, SOFA score, and CRP level (Table 3)

In the univariate analysis, patients with SARS due to causes other than COVID-19 compared with those with SARS due to COVID-19 were 1.5 times more likely to die (OR 2.528, 95% CI 1.642–3.892, p < 0.001). The six parameters of glycemic abnormalities emerged as risk factors for ICU mortality within 30 days when adjusted for COVID-19 alone. Similarly, after adjustment for COVID-19, the odds of death increased with higher APACHE II scores, SOFA scores, CRP values, age, prior history of diabetes, and development of nosocomial infection (Table 4)

In the multivariate explanatory models of mortality, the parameters of glycemic abnormalities: highest glucose level at admission, mean and highest glucose levels during ICU stay, glucose variability, and percentage of days with hyperglycemia, remained independent risk factors for mortality even after adjustment for variables related to mortality, including COVID-19, history of diabetes, nosocomial infection, age, APACHE II score, SOFA score, and CRP level. In this same context, COVID-19 also remained an independent risk factor for mortality (Table 5). The occurrence of hypoglycemia (< 70 mg/dL) during ICU stay was no longer statistically significant in relation to mortality when adjusted for confounders (Additional file: Table S3)

The crude relationship between mean glucose level during ICU stay intervals and mortality within 30 days in the ICU is as show in Additional file 1: Figure S1. An U-shaped relationship was identified between mean glucose and mortality in the SARS without COVID-19 cohort, while in the SARS due to COVID-19 cohort the mortality does appears to increase in a linear fashion with mean glucose levels (Additional file 1: Figure S1)

## Discussion

The findings of the study demonstrated that in a population of patients admitted to the ICU with SARS and suspected COVID-19, those in whom the diagnosis of COVID-19 was confirmed, compared with those in whom this diagnosis was refuted, had higher glycemic load, represented by higher glucose level at admission, higher glucose level and higher mean glucose level during ICU stay, mean glucose variability, and higher percentage of days with hyperglycemia during ICU stay. However, this association between COVID-19 and glycemic abnormalities was no longer statistically significant after being adjusted for confounding factors. On the other hand, APACHE II scores, SOFA scores, use of corticosteroids, and presence of nosocomial infection remained associated with the parameters representing dysglycemia, which is consistent with findings in critically ill patients [1]. The development of dysglycemia, independent of the patient’s preadmission glucose level, has been frequently reported in hospitalized patients with severe COVID-19 and is associated with worse outcomes [6, 11]. Many studies have analyzed the relationship between hyperglycemia and severe COVID-19, but no study has evaluated hyperglycemia and other glycemic abnormalities in patients with SARS due to COVID-19 compared with contemporary controls. Our retrospective cohort of patients admitted to the ICU addressed this issue specifically. The objective of our study was to assess whether dysglycemias were directly associated with COVID-19 or if they were more common in these patients because they were more seriously ill. Another objective was to evaluate whether COVID-19 and dysglycemias were each independently related to mortality in these patients.

Observational studies have shown associations of severe hyperglycemia and increased glucose variability with poor outcomes in patients with COVID-19 [8, 24], although the causality of these associations remains uncertain since hyperglycemia and insulin resistance are closely related to disease severity [25]. The hypothesis that COVID-19 is specifically related to hyperglycemia and recent-onset diabetes [6] has even led to a search for effects directly mediated by the virus on the function and survival of insulin-producing beta cells and insulin resistance [26].

Our study comparing patients with SARS due to COVID-19 against patients with SARS due to other causes suggests that the dysglycemia in patients with COVID-19 was not directly associated with the SARS-CoV-2 infection as previously proposed [27], but is due to greater insulin resistance, probably secondary to higher levels of counterregulatory hormones and cytokines and the use of medications, although beta cell dysfunction secondary to prolonged hyperglycemia, inflammation, and/or low beta-cell reserve cannot be ruled out [25].

Compared with patients who do not meet diabetes or uncontrolled hyperglycemia criteria, patients with theses abnormalities are significantly older [9, 17], sometimes with a higher percentage of male [9]. Both daily SOFA and APACHE II have been considered mortality predictors in critically ill COVID-19 patients [18]. Steroids have been known for increasing blood sugar by increasing the hepatic gluconeogenesis or production of glucose from the liver and by enhancing the effect of counter regulatory hormones, among other mechanisms [21, 23]. Elevations in the levels of inflammatory cytokines as seen in COVID-19 further worsen insulin resistance [19], and levels of inflammatory markers and markers of COVID-19 severity, including C-reactive protein, have been found to be higher in patients with newly diagnosed diabetes, compared to those with pre-existing DM [20].

Regarding the relationship between dysglycemia and mortality, our study showed that the same parameters representing glycemic load and related to COVID-19 are independent risk factors for mortality in up to 30 days in the ICU, even when adjusted for factors also related to mortality. Hyperglycemia has been linked to a higher risk of mortality and, together with cardiac injury and use of high doses of corticosteroids [10], is considered an independent predictor of mortality in hospitalized patients with COVID-19 [28], which has been confirmed in the present study. Other retrospective studies in patients hospitalized with COVID-19 have also shown associations between hyperglycemia and worse prognosis [7, 29] independent of pre-existing diabetes [8], and even higher risk of mortality in patients with COVID-19 without diabetes compared with those with diabetes. The finding that the variables related to glycemic load and length of ICU stay remained associated with mortality suggests that effective methods to detect and treat dysglycemia may reduce the impact of these glucose abnormalities in patients with COVID-19 and improve their outcome [30, 31].

Due to the observational nature of the study, not all data were available for all patients, and we were unable to control for all factors possibly interfering with glucose levels, including the daily amount of insulin units used to correct hyperglycemia. However, all included patients received the same standard capillary blood glucose correction protocol, with a blood glucose target < 140 mg/dL. Also, since critically ill patients may present with hemodynamic failure, the agreement between venous and capillary glucose levels may be lower than expected. But even though we considered capillary and venous glucose levels indistinctively, the differences in dysglycemia between groups could not be attributed only to this fact. Other limitations of our study include the information about presumed obesity, which was subjectively determined by the attending physician, and the fact that the absence of a history of diabetes does not exclude the possibility of undiagnosed diabetes. As an important strength, the findings of our study, with a longitudinal follow-up of a large number of patients with SARS, offer potentially useful information about the influence of COVID-19 on glucose levels in this population and its independent impact on mortality.

## Conclusion

In conclusion, our study demonstrated that patients with SARS and COVID-19 have more frequent dysglycemia and higher mortality than patients with SARS due to other causes. However, the dysglycemias were not independently associated with COVID-19, but rather related to disease severity, inflammatory markers and the use of glucocorticoids.

## Electronic supplementary material

Below is the link to the electronic supplementary material.

Supplementary Material 1

## Data Availability

The dataset supporting the conclusions of this article is available in the Zenodo.org repository, with the link 10.5281/zenodo.6959376.

## Abbreviations

ICU: Intensive care unit
ICUs: Intensive care units
SARS: Severe acute respiratory syndrome
SUS: Brazilian Unified Health System
RT-PCR: Real-time polymerase chain reaction
CT: Computed tomography
APACHE: Acute Physiology and Chronic Health Evaluation
SOFA: Sequential Organ Failure Assessment
CRP: C-reactive protein (mg/L)
eCoef.: Exponentiated linear regression coefficients
Coef.: Regression coefficients
OR: Odds ratio
BIC: Bayesian information criterion
VAD: Vasoactive drug
IMV: Invasive mechanical ventilation
