# Supplementary material for: Dysglycemias in patients admitted to ICUs with severe acute respiratory syndrome due to COVID-19 versus other causes - a cohort study
Source: BMC Pulm Med. 2023 May 16;23:173. doi: 10.1186/s12890-023-02439-y (PMC10187505; doi:10.1186/s12890-023-02439-y)

# ADDITIONAL FILE 1

## Dysglycemias in patients admitted to ICUs with severe acute respiratory syndrome due to COVID-19 versus other causes – A cohort study

Rosângela Roginski Réa[1,2], Rafaella Stradiotto Bernardelli[2-3], Amanda Christina Kozesinski-Nakatani[2,4], Marcia Olandoski[3], Marcelo José Martins-Junior[2], Mirella Cristine de Oliveira[2,5], Álvaro Réa-Neto[1,2].

## ADDITIONAL FILE 1 CONTENTS

**Table S1**. Table S1 - Characteristics of patients from each of the eight participating sites (hospitals)………………………………………………………………………………………………………… 2

**Table S2**. Influence of COVID-19 on occurrence of hypoglycemia (<70 mg/dL) during ICU stay unadjusted and adjusted for the variables sex, history of diabetes, presumed obesity, age, APACHE II scores, SOFA scores, CRP levels, and corticosteroid use………… 4

**Table S3**. Adjusted model explaining mortality within 30 days in ICU, including occurrence of hypoglycemia……………………………………………………………………………………. 5

**Figure S1** Association between mean glucose levels and 30 days ICU mortality for overall cohort and for each group: COVID-19 and Non-COVID-19………………………………………… 6

Table S1 - Characteristics of patients from each of the eight participating sites (hospitals).

| | HOSPITALS (sites) | | | | | | | |
|---|---|---|---|---|---|---|---|---|
| **VARIABLES** | HR (n=210) | HT (n=198) | HSC (n=160) | HIM (n=116) | HVB (n=80) | HMC (n=74) | HN (n=32) | INC (n=1) |
| **Baseline** | | | | | | | | |
| Diagnoses | | | | | | | | |
| Non-COVID-19 | 13 (6.2) | 27 (16.1) | 12 (7.5) | 43 (37.1) | 18 (22.5) | 15 (20.3) | 9 (28.1) | 1 (100) |
| COVID-19 | 197 (93.8) | 141 (83.9) | 148 (92.5) | 73 (62.9) | 62 (77.5) | 59 (79.7) | 23 (71.9) | 0 (0) |
| Age (years) | 59.1 ± 14.7 | 57.2 ± 15.7 | 65 ± 16.5 | 64 ± 15.8 | 58 ± 17.7 | 62.4 ± 20 | 67.6 ± 16.5 | 80 ± 0 |
| Male sex | 111 (52.9) | 99 (58.9) | 91 (56.9) | 59 (50.9) | 50 (62.5) | 45 (60.8) | 14 (43.8) | 1 (100) |
| History of diabetes | 44 (26.2) | 72 (34.3) | 56 (35) | 40 (34.5) | 21 (26.3) | 18 (24.3) | 8 (25) | 0 (0) |
| Presumed obesity | 70 (41.7) | 88 (41.9) | 19 (11.9) | 30 (25.9) | 18 (22.5) | 20 (27) | 5 (15.6) | 0 (0) |
| Charlson Comorbidity Index | 1 (0 - 1) | 1 (0 - 1) | 1 (0.5 - 2) | 1 (0 - 2) | 1 (0 - 2) | 1 (0 - 2) | 1 (0 - 1) | 3 (3 - 3) |
| **ICU admission status** | | | | | | | | |
| APACHE II score in the first 24hrs in ICU | 11.5 (7 - 18) | 13 (8 - 20) | 19 (12 - 28) | 11 (7 - 17) | 11 (5 - 16.5) | 14 (10 - 18) | 11.5 (6 - 17.5) | 20 (20 - 20) |
| SOFA score in the first 24 hrs in ICU | 3 (2 - 6) | 3 (2 - 6) | 5 (3 - 7) | 4 (3 - 6) | 3 (2 - 5) | 3 (2 - 6) | 3 (2 - 4.5) | 13 (13 - 13) |
| CRP level in the first 24 hrs in the ICU[a] | 110 (57 - 173.5) | 122 (71.5 - 183.5) | 138.9 (84.1 - 199) | 109 (30.7 - 177.5) | 79.5 (31.5 - 134.5) | 108.5 (52 - 165) | 77 (51 - 129) | 122 (122 - 122) |
| **Related to glycemic abnormalities in the ICU** | | | | | | | | |
| Highest glucose value at ICU admission[b] | 172.5 (132 - 255) | 169.5 (139.5 - 219) | 146 (113.5 - 222) | 158 (122 - 225) | 152.5 (114.5 - 201) | 138 (112 - 195) | 146.5 (116 - 169) | 108 (108 - 108) |
| Highest glucose value during ICU stay | 251.5 (180 - 388) | 242.5 (175 - 387.5) | 226 (154 - 366.5) | 254.5 (167 - 376.5) | 203.5 (136.5 - 363.5) | 236 (161 - 361) | 176 (150 - 298.5) | 153 (153 - 153) |
| Mean glucose value during ICU stay | 156.2 (132.2 - 218.1) | 144.4 (123.4 - 195) | 138.5 (111.7 - 183.1) | 152.2 (122.9 - 213.6) | 137.4 (110.9 - 173.3) | 148.8 (123 - 195.8) | 124.4 (106.9 - 171.5) | 130.5 (130.5 - 130.5) |
| Mean glucose variability (CV %) during ICU stay[c] | 23.4 (18.4 - 34.2) | 25.3 (18.8 - 36.3) | 35.4 (24.2 - 51.3) | 31.6 (22.3 - 43.1) | 25.6 (17.8 - 36.3) | 31.5 (24.8 - 42.9) | 25.1 (20 - 37.7) | . (. - .) |

| | | | | | | | | |
|---|---|---|---|---|---|---|---|---|
| % of days with hyperglycemia (≥180 mg/dL) | 50 (7.1 - 90) | 34.2 (0 - 81.8) | 33.3 (0 - 84.5) | 50 (0 - 87.5) | 25 (0 - 65.2) | 23.2 (0 - 76.5) | 0 (0 - 47.7) | 0 (0 - 0) |
| Occurrence of hypoglycemia (<70 mg/dL) during ICU stay | 34 (16.2) | 42 (25) | 77 (48.1) | 36 (31) | 14 (17.5) | 10 (13.5) | 5 (15.6) | 0 (0) |
| **Related to progression within 30 days of ICU stay** | | | | | | | | |
| Nosocomial infection | 41 (24.4) | 55 (26.2) | 20 (12.5) | 15 (12.9) | 19 (23.8) | 14 (18.9) | 5 (15.6) | 0 (0) |
| Mean SOFA score during ICU stay | 4 (2.2 - 6.4) | 3.6 (2 - 6.6) | 6 (3.3 - 8.5) | 5.3 (3.3 - 7.3) | 3 (2 - 5.8) | 3 (2 - 5.6) | 2.8 (1.9 - 7) | 14 (14 - 14) |
| Mean CRP level during ICU stay[a] | 133.5 (74.7 - 185.7) | 125.2 (61.4 - 179.2) | 154.2 (82.9 - 202.7) | 111.2 (60.5 - 181) | 79.5 (43.9 - 132.6) | 105.7 (66.7 - 152.8) | 93.3 (46 - 143.8) | 210 (210 - 210) |
| Use of corticosteroid during ICU stay | 96 (57.1) | 151 (71.9) | 102 (63.8) | 109 (94) | 41 (51.3) | 39 (52.7) | 13 (40.6) | 0 (0) |
| ICU length of stay | 8 (4 - 20) | 6 (3 - 10) | 5 (2 - 9) | 6 (4 - 12) | 4 (2 - 10) | 7 (4 - 15) | 5 (2.5 - 10) | 1 (1 - 1) |
| 30 days ICU mortality | 73 (34.8) | 48 (28.6) | 84 (52.5) | 66 (56.9) | 22 (27.5) | 17 (23) | 9 (28.1) | 1 (100) |

Data are expressed as number (percent), mean ± standard deviation, or median (first quartile - third quartile).

Abbreviations: APACHE II, Acute Physiology and Chronic Health Evaluation II; SOFA, Sequential Organ Failure Assessment; CRP, C-reactive protein (mg/L); VAD: vasoactive drug; IMV: invasive mechanical ventilation. The glucose values are shown as mg/dL; hrs: hours; CV, Coefficient of variation.

Abbreviations for the names of the hospitals: HT, Hospital do Trabalhador; HR, Hospital de Reabilitação; HSC, Hospital Santa Casa de Curitiba; IMP, Instituto de Medicina do Paraná; HVB, Hospital Vita Batel; HMC, Hospital Marcelino Champagnat; HN, Hospital das Nações; and INC, Instituto de Neurologia de Curitiba.

[a] 2 missing data in the HR; 1 missing data in the HSC; 1 missing data in the HN.

[b] 4 missing data in the IM.

[c] 10 missing data in the HT; 16 missing data in the HR; 2 missing data in the HSC; 4 missing data in the IM; 2 missing data in the HVB; 36 missing data in the HMC; 5 missing data in the HN; and 1 missing data in the INC.

**Table S2.** Influence of COVID-19 on occurrence of hypoglycemia (<70 mg/dL) during ICU stay unadjusted and adjusted for the variables sex, history of diabetes, presumed obesity, age, APACHE II scores, SOFA scores, CRP levels, and corticosteroid use.

| **Factors associated with the occurrence of hypoglycemia (<70 mg/dL) during ICU stay** | **n[$]** | **OR (95% CI) unadjusted†** | **p value[§]** | **n[$$]** | **OR (95% CI) adjusted††** | **p value[§]** |
|---|---|---|---|---|---|---|
| COVID-19 [a] | 841 | 1.084 (0.711 - 1.654) | 0.707 | 837 | 0.856 (0.539 - 1.36) | 0.510 |
| Male sex [b] | 841 | 0.843 (0.618 - 1.149) | 0.279 | | - | - |
| History of diabetes [c] | 841 | 1.941 (1.406 - 2.678) | 0.000 | | 1.906 (1.339 - 2.712) | 0.000 |
| Presumed obesity [d] | 841 | 0.764 (0.54 - 1.083) | 0.130 | | 0.7 (0.475 - 1.03) | 0.070 |
| Charlson Comorbidity Index | 841 | 1.188 (1.066 - 1.324) | 0.002 | | - | - |
| Age | 841 | 1.017 (1.007 - 1.027) | 0.001 | | 1 (0.988 - 1.011) | 0.972 |
| APACHE II scores | 841 | 1.048 (1.032 - 1.065) | 0.000 | | 1.011 (0.991 - 1.031) | 0.288 |
| Nosocomial infection [e] | 841 | 2.952 (2.069 - 4.211) | 0.000 | | 2.178 (1.478 - 3.209) | 0.000 |
| Mean SOFA during ICU stay | 841 | 1.269 (1.202 - 1.339) | 0.000 | | 1.191 (1.103 - 1.287) | 0.000 |
| Mean CRP during ICU stay | 837 | 1.006 (1.004 - 1.008) | 0.000 | | 1.001 (0.999 - 1.004) | 0.342 |
| Use of corticosteroid during ICU stay [f] | 841 | 1.187 (0.854 - 1.65) | 0.307 | | - | - |
| | | | | | BIC: 907.242‡ | |

[$] number of cases (n) included in each univariate model.

[$$] number of cases (n) included in the multivariate model; [§] Wald test p value, results <0.05 indicate statistical significance.

[§]Wald test p value, p < 0.05 indicates statistical significance.

[‡] Bayesian information criterion (BIC) of the multivariate generalized linear model, representative of the quality of the model given its explanatory potential, with the lower the BIC, the more the model is able to explain the dependent variable.

† Odds ratio (OR) and 95% confidence interval (95% IC) of the generalized linear model with univariate binary logistic distribution.

†† Odds ratio (OR) and 95% confidence interval (95% IC) of the generalized linear model with multivariate binary logistic distribution.

**Table S3 -** Adjusted model explaining mortality within 30 days in ICU, including occurrence of hypoglycemia.

| **Multivariate explanatory models of mortality in the overall cohort** | **n[#]** | **OR (95% CI) adjusted*** | **p value [§]** | **BIC ‡** |
|---|---|---|---|---|
| Occurrence of hypoglycemia (<70 mg/dL) during ICU stay (ref: no hypoglycemia) | 837 | 0.719 (0.422 - 1.227) | 0.227 | 523.057 |
| COVID-19 (ref: non-COVID-19) | | 2.865 (1.467 - 5.597) | 0.002 | |
| History of diabetes (ref: no history) | | 1.169 (0.699 - 1.958) | 0.552 | |
| Age | | 1.016 (1 - 1.033) | 0.057 | |
| APACHE II score | | 1 (0.97 - 1.031) | 0.983 | |
| Nosocomial infection (Ref: absent) | | 1.247 (0.738 - 2.109) | 0.409 | |
| Mean SOFA score | | 2.458 (2.104 - 2.871) | 0.000 | |
| Mean CRP level | | 1.009 (1.006 - 1.013) | 0.000 | |

Abbreviations: APACHE II, Acute Physiology and Chronic Health Evaluation II; SOFA, Sequential Organ Failure Assessment; CRP, C-reactive protein (mg/L).

[#] Case numbers included in the model

* Odds ratio (OR) and 95% confidence interval (95% CI) for mortality within 30 days in the ICU, generalized linear models with multivariate binary logistic regression.

[§] Wald test p value, with values <0.05 indicating statistical significance.

[‡] Bayesian information criterion (BIC) of the multivariate generalized linear model representing the quality of the model given its explanatory potential; the lower the BIC, the more the model is able to explain the dependent variable.

**Figure S1 -** Association between mean glucose levels and 30 days ICU mortality for overall cohort and for each group: COVID-19 and Non-COVID-19

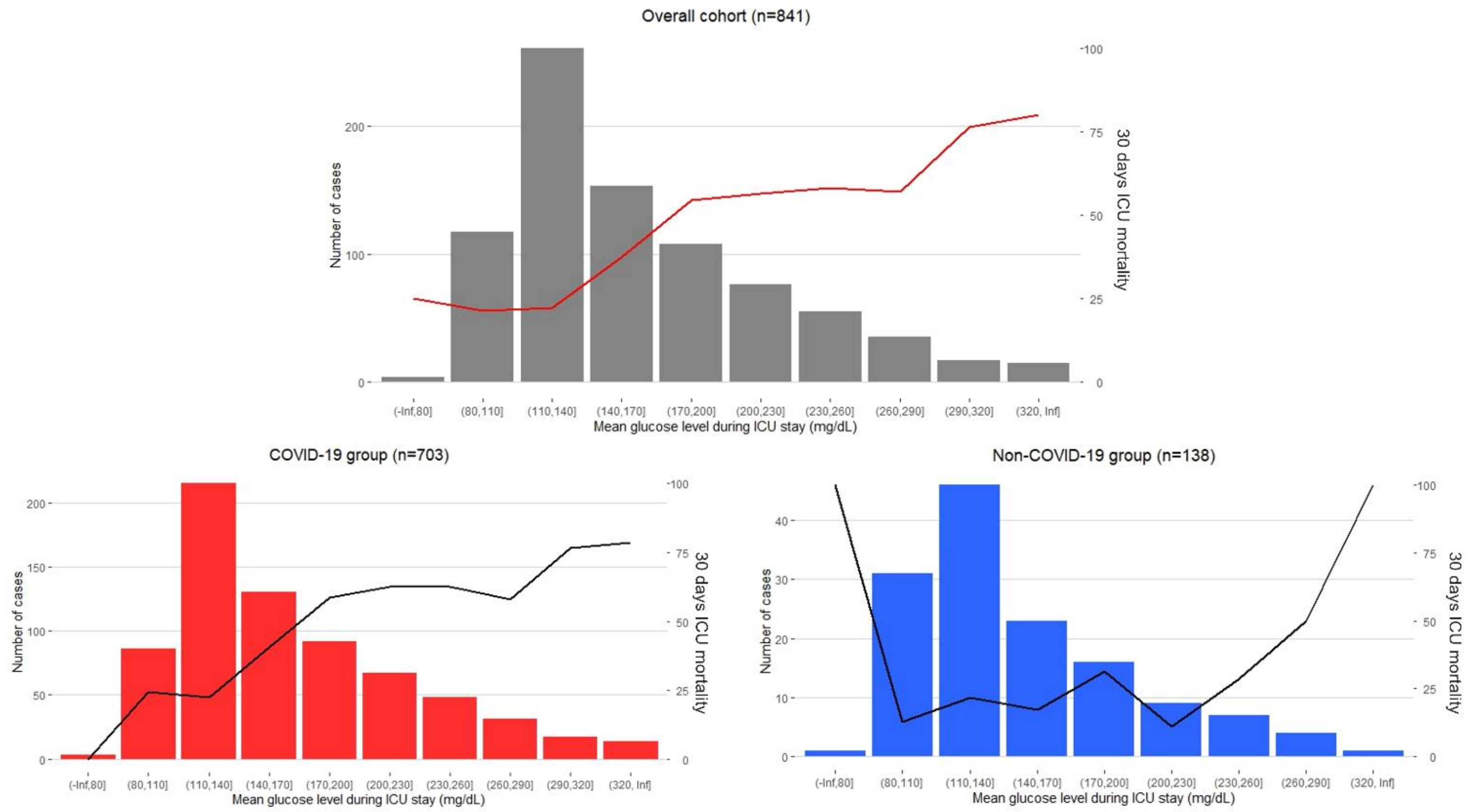

Supplement: Supplementary file 1 — Supplementary Material 1 [file 12890_2023_2439_MOESM1_ESM.pdf]
